# An outbreak vector-host epidemic model with spatial structure: the 2015–2016 Zika outbreak in Rio De Janeiro

**DOI:** 10.1186/s12976-017-0051-z

**Published:** 2017-03-27

**Authors:** W. E. Fitzgibbon, J. J. Morgan, G. F. Webb

**Affiliations:** 1 Department of Mathematics, University of Houston, Houston, 77204 TX USA; 2 Department of Mathematics, Vanderbilt University, Nashville, 37240 TN USA

**Keywords:** Criss-cross dynamics, Local reproduction number, Zika epidemic

## Abstract

**Background:**

A deterministic model is developed for the spatial spread of an epidemic disease in a geographical setting. The disease is borne by vectors to

susceptible hosts through criss-cross dynamics. The model is focused on an outbreak that arises from a small number of infected hosts imported into a subregion of the geographical setting. The goal is to understand how spatial heterogeneity of the vector and host populations influences the dynamics of the outbreak, in both the geographical spread and the final size of the epidemic.

**Methods:**

Partial differential equations are formulated to describe the spatial interaction of the hosts and vectors. The partial differential equations have reaction-diffusion terms to describe the criss-cross interactions of hosts and vectors. The partial differential equations of the model are analyzed and proven to be well-posed. A local basic reproduction number for the epidemic is analyzed.

**Results:**

The epidemic outcomes of the model are correlated to the spatially dependent parameters and initial conditions of the model. The partial differential equations of the model are adapted to seasonality of the vector population, and applied to the 2015–2016 Zika seasonal outbreak in Rio de Janeiro Municipality in Brazil.

**Conclusions:**

The results for the model simulations of the 2015–2016 Zika seasonal outbreak in Rio de Janeiro Municipality indicate that the spatial distribution and final size of the epidemic at the end of the season are strongly dependent on the location and magnitude of local outbreaks at the beginning of the season. The application of the model to the Rio de Janeiro Municipality Zika 2015–2016 outbreak is limited by incompleteness of the epidemic data and by uncertainties in the parametric assumptions of the model.

## Background

The Zika virus is a mosquito borne flavivirus that was first isolated in Uganda in 1947 [1]. Subsequently, it has become prevalent in parts of Africa, Asia, and Central and South America. The geographic distribution of the virus has been steadily increasing since 2015 and its further geographic spread to additional countries that are home to competent mosquito vectors is highly probable. As of September 15, 2016, the World Health Organization reports that local circulation of the virus has been reported by 72 countries and territories. Although there have been reports of transmissions through sexual contact [2], Zika virus appears to be primarily spread through the human population through bites from *Aedes* mosquitos. The virus incubates in a human host over an asymptomatic period lasting from three to twelve days and once fully developed, the virus disease persists for about a week. It is characterized by low grade fever, rash, joint pain, and conjunctivitis (red eyes). Typically it is mild and seldom requires hospitalization. However the virus has two severe complications which make it a menace to public health. The virus has been linked to an increased risk of Guillian-Barre syndrome which is a severe autoimmune disorder [3]. Perhaps even more serious is its linkage to microcephaly birth defects in newborn babies [4].

Zika epidemics are both year-round and seasonal, dependent upon the year-round prevalence or seasonality of the resident mosquito populations. A recent study [5] describes in detail the potential spread of Zika epidemics into African and Asian-Pacific regions by the importation of infected people. The generation of Zika epidemics by the importation of infected people into year-round or seasonal environments is a major public health concern. Recent mathematical models have been developed to understand these concerns [6–13]. We develop a model that describes both year-round and seasonal host-vector epidemic population dynamics in a geographical region. The disease is borne by vectors to susceptible hosts through criss-cross dynamics in a region of spatially distributed vectors and hosts. The epidemic outbreak begins with the arrival of a small number of viremic hosts in one or more locales in which the disease is not yet present. Our goal is to aid understanding of how the introduction of a small number of infected hosts, in a specific location in a geographic region, will result in a dissipated or a sustained epidemic. The focus of the study is examine the influence of spatial effects on these possible outcomes.

We formulate a criss-cross reaction-diffusion partial differential equations model to describe the spatial evolution of an epidemic. Criss-cross reaction-diffusion models for the circulation of disease between vectors and hosts have been used to describe the spatial spread of malaria [14], the spatial spread of Dengue outbreaks [15, 16], and the spatial spread of other diseases by many authors [17–24]. We apply our model to the 2015–2016 Zika seasonal outbreak in the urban area of Rio de Janeiro Municipality in Brazil. We numerically simulate the model to analyze varied scenarios of Zika seasonal epidemics in Rio de Janeiro, dependent upon the input of local spatial outbreaks at the beginning of the season and the time-limitation of seasonality.

## Methods

The geographical region is denoted by *Ω*⊂*R* ^2^. The background population of uninfected and susceptible hosts in *Ω* has geographic density *H*_*u*_(*x*,*y*), which is assumed unchanging in time in the demographic and epidemic context of the outbreak. Thus, the model is viewed as applicable to an early phase of the epidemic, during which the epidemic does not alter the local geographic and demographic population structure of hosts. The model consists of the following compartments:

- The density of infected hosts *H*_*i*_(*t*,*x*,*y*) at time *t* at (*x*,*y*)∈*Ω*, with initial condition *H*_*i*0_(*x*,*y*).
- The density of uninfected vectors *V*_*u*_(*t*,*x*,*y*) at time *t* at (*x*,*y*)∈*Ω*, with initial condition *V*_*u*0_(*x*,*y*).
- The density of infected vectors *V*_*i*_(*t*,*x*,*y*) at time *t* at (*x*,*y*)∈*Ω*, with initial condition *V*_*i*0_(*x*,*y*).

### Equations of the model

The equations of the model in the case that transmission from vectors to hosts is year-round are

1 $$\begin{array}{@{}rcl@{}}  \frac{\partial}{\partial t} H_{i}(t,x,y) &=& \nabla \cdot \delta_{1}(x,y) \nabla H_{i}(t,x,y) - \lambda(x,y) \, H_{i}(t,x,y)  \end{array} $$

2 $$\begin{array}{@{}rcl@{}} &&+ \sigma_{1}(x,y) \, H_{u}(x,y) \, V_{i}(t,x,y),  \\ \frac{\partial}{\partial t} V_{u} (t,x,y) &=& \nabla \cdot \delta_{2}(x,y) \nabla V_{u}(t,x,y) - \sigma_{2}(x,y) V_{u}(t,x,y) H_{i}(t,x,y)  \end{array} $$

3 $$\begin{array}{@{}rcl@{}} &&+ \beta(x,y) \left(V_{u}(t,x,y) + V_{i}(t,x,y) \right)  \\ &&- \mu(x,y) \left(V_{u}(t,x,y) + V_{i}(t,x,y) \right) V_{u}(t,x,y),  \\ \frac{\partial}{\partial t} V_{i} (t,x,y) &=& \nabla \cdot \delta_{2}(x,y) \nabla V_{i}(t,x,y) + \sigma_{2}(x,y) V_{u}(t,x,y) H_{i}(t,x,y)  \end{array} $$

$$\begin{array}{@{}rcl@{}} & & - \mu(x,y) \left(V_{u}(t,x,y) + V_{i}(t,x,y) \right) V_{i}(t,x,y).  \end{array} $$

In addition, the following boundary and initial conditions are satisfied:

$$\frac{\partial}{\partial \eta} H_{i}(t,x,y) = 0, \, \frac{\partial}{\partial \eta} V_{u}(t,x,y) = 0, \, \frac{\partial}{\partial \eta} V_{i}(t,x,y) = 0, \, (x,y) \in \partial \Omega, \, t >0, $$

$$H_{i}(0,x,y) = H_{i0}(x,y), \, V_{u}(0,x,y) = V_{u0}(x,y), \, V_{i}(0,x,y) = V_{i0}(x,y), \, (x,y) \in \Omega. $$

The spatially dependent parameters of the model are as follows: *λ*(*x*,*y*) is the loss rate of the infected host population (due to recovery or other removal). *β*(*x*,*y*) is the breeding rate of the vector population. *μ*(*x*,*y*) is the loss rate of the vector population due to environmental crowding. *σ*_1_(*x*,*y*) is the transmission rate of uninfected hosts and *σ*_2_(*x*,*y*) is the transmission rate of uninfected vectors. The transmission terms for both hosts and vectors are assumed to be of density-dependent form, rather than frequency-dependent form [25]. A comparison of the two forms for spatially dependent models is given in [26]. Since we assume, during the early phase of the epidemic, that the populations of infected hosts (infected vectors) are relatively small fractions of the populations of uninfected hosts (uninfected vectors), the two forms are essentially the same. *δ*_1_(*x*,*y*) and *δ*_2_(*x*,*y*) are the diffusion rates of the infected hosts and infected vector populations, respectively.

In the Appendix we prove the well-posedness of the model.

### The local basic reproduction number

Define the local basic reproduction number of the model (1), (2), (3) as follows:

$$R_{0}(x,y) = \frac{\sigma_{1}(x,y) \sigma_{2}(x,y) H_{u}(x,y)}{\lambda(x,y) \mu(x,y)}. $$

*R*_0_(*x*,*y*) is interpreted as the average number of new cases generated by a single case at a given location (*x*,*y*) in *Ω*. An analysis of local reproduction numbers for spatially dependent models is given in [27] and in [28]. Our motivation for this definition is the basic reproduction number *R*_0_ of the spatially independent model (Appendix). Simulations of the spatially dependent model show the following behavior: (1) If *R*_0_(*x*,*y*)<1 everywhere in *Ω*, then the populations of both infected hosts and infected vectors extinguish, and the populations converge to the disease free equilibrium. (2) If *R*_0_(*x*,*y*)>1 in some subregion *Ω*_0_⊂*Ω*, then the populations of both infected hosts and infected vectors may converge from an initial local outbreak to an endemic equilibrium in *Ω*, even if the average value of *R*_0_(*x*,*y*) in all of *Ω* is <1.

### Equations of the model when the vector population is seasonal

If the vector population is seasonal, then equations of the model must be modified to account for seasonality. We assume that the vector population breeding term *β*(*t*,*x*,*y*) is dependent on time. We assume that in addition to the vector loss parameter *μ*(*x*,*y*) corresponding to carrying capacity, there is a time-independent vector loss term *μ*_1_(*x*,*y*), corresponding to the average vector life-span 1/*μ*_1_(*x*,*y*). The modified equations are

4 $$\begin{array}{@{}rcl@{}}  \frac{\partial}{\partial t} V_{u} (t,x,y) &=& \nabla \cdot \delta_{2}(x,y) \nabla V_{u}(t,x,y) - \sigma_{2}(x,y) V_{u}(t,x,y) H_{i}(t,x,y)  \end{array} $$

5 $$\begin{array}{@{}rcl@{}} &&+ \beta(t,x,y) \left(V_{u}(t,x,y) + V_{i}(t,x,y) \right) - \mu_{1}(x,y) V_{u}(t,x,y)  \\ & & - \mu(x,y) \left(V_{u}(t,x,y) + V_{i}(t,x,y) \right) V_{u}(t,x,y),  \\ \frac{\partial}{\partial t} V_{i} (t,x,y) &=& \nabla \cdot \delta_{2}(x,y) \nabla V_{i}(t,x,y) + \sigma_{2}(x,y) V_{u}(t,x,y) H_{i}(t,x,y)  \end{array} $$

$$\begin{array}{@{}rcl@{}} & & - \mu(x,y) \left(V_{u}(t,x,y) + V_{i}(t,x,y) \right) V_{i}(t,x,y)  \\ & & - \mu_{1}(x,y) V_{i}(t,x,y).  \end{array} $$

### The 2015–2016 Zika outbreak in Rio de Janeiro municipality

**Fig. 1:**
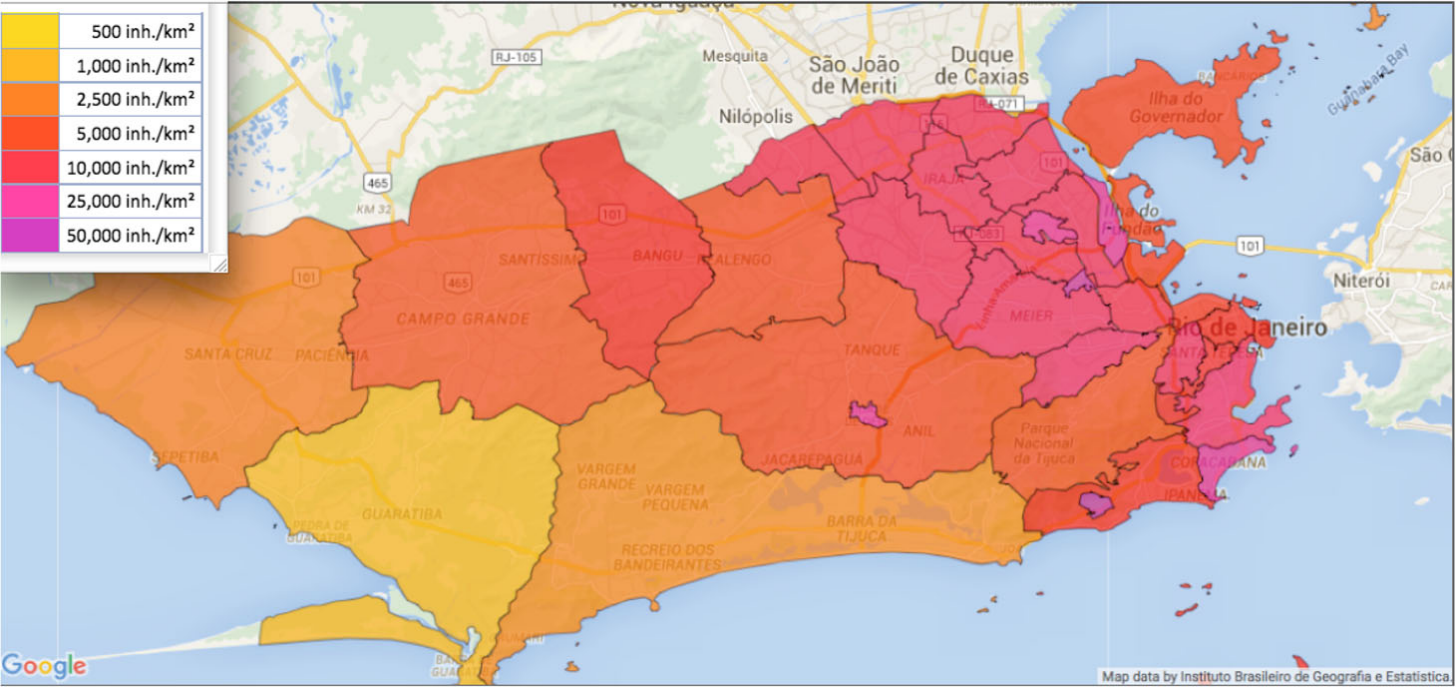
Rio de Janeiro Municipality sub-districts. The sub-district population densities range from 1,000 to 50,000 inhabitants per square kilometer. The Municipality is approximately 50 kilometers east-west by 20 kilometers north-south, with the highest population density in the eastern region. The total population is approximately 6,000,000. (Source: http://www.citypopulation.de/php/brazil-rio. php)

We apply the model (1), (4), (5) to the 2015–2016 Zika epidemic in Rio de Janeiro, Brazil. The host population are the people in the Municipality, which in 2016 is approximately 6,000,000, in a geographical region of approximately 1,200 square kilometers (Source: *Instituto Brasileiro de Geografia e Estatistica*). The vector population is the female *Aedes aegypti* mosquito. The Municipality comprises 33 sub-districts, with population densities ranging from 1,000 to 50,000 inhabitants per square kilometer (Fig. 1). The period November through July can be viewed as the seasonal Zika transmission period of the epidemic in the Municipality.

A small number of cases were recorded in the Municipality into the summer of 2015, with the highest number of cases in the eastern region of the Municipality [29, 30]. The Brazilian Health Ministry [31] reported that Rio de Janeiro State (population approximately 16,000,000) registered a count of 60,176 cumulative cases from January 1, 2016 to August 13, 2016 (incidence of approximately 364 cases per 100,000 inhabitants). In [32] the weekly case data for Rio de Janeiro Municipality is given from November 1, 2015 through April 10, 2016, during which time the reporting of cases became mandatory. The cumulative number of reported cases in the Municipality during this period was 25,400 [32] (incidence of approximately 423 cases per 100,000 inhabitants).

### Parameterization of the Rio de Janeiro model

We simulate the model (1), (4), (5) for Rio de Janeiro Municipality with some parameters assumed. The available epidemic data used for comparison to our simulations for the Rio de Janeiro Municipality 2015–2016 Zika outbreak is very limited. Further, the number of unreported cases, necessarily unknown, is a limitation of the applicability of the model for this application. A more precise fitting of parameters *μ*, *σ*, and *β* requires much higher data accuracy specific to the Zika epidemic in the Municipality. Our purpose is to provide a qualitative description of a typical vector-borne epidemic spatial outbreak, and our simulation of this particular outbreak, with its limitations on parameterization, serves this purpose.

**Fig. 2:**
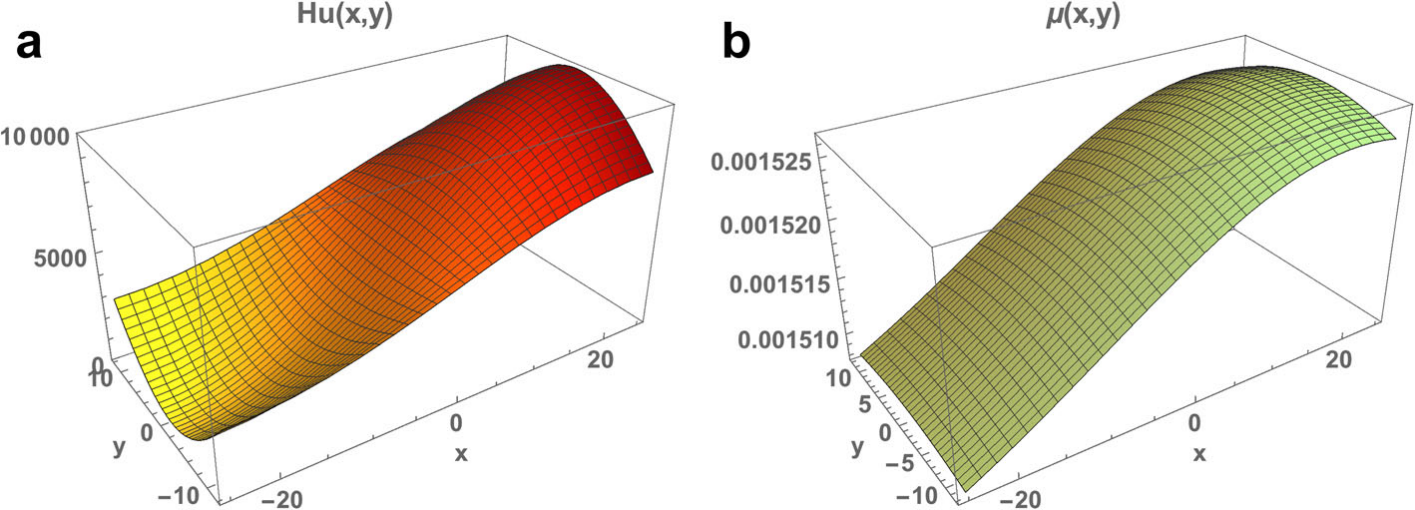
**a** The population of susceptible people *H*_*u*_(*x*,*y*) in Rio de Janeiro Municipality, which agrees approximately with the geographical population density in Fig. 1. **b** The spatially dependent mosquito loss function *μ*(*x*,*y*), which is higher in locations of higher population density due to mosquito control measures

Explanations for our assumptions on specific parameter values are as follows: The time units for our simulations are weeks. The spatial units are kilometers and *Ω*=(−25,25)×(−12,12). The boundary conditions for *Ω* are a reasonable simplification of the costal boundaries and the less populated northern boundary of Rio de Janeiro Municipality. The average length of the infectious period of infected people is approximately 1 to 2 weeks and we set *λ*(*x*,*y*)=1.0 [33, 34]. The average lifespan of female *Aedes aegypti* mosquitoes is approximately two weeks in an urban environment [35, 36], and we set *μ*_1_(*x*,*y*)=0.5. The total uninfected host population is approximately approximately 6,000,000, with geographical density function *H*_*u*_(*x*,*y*)=50.0+10^2^ (1.0+ sin(0.02*π* *x*) cos(0.03*π* *y*)) (Fig. 2 a), which corresponds approximately to the population density distribution in Fig. 1.

We set the density dependent mosquito loss function *μ*(*x*,*y*)=0.0015(1.0+100 *g* *a* *u* *s* *s*(20.0,30.0,*x*)×*g* *a* *u* *s* *s*(0.0,30.0,*y*)) (Fig. 2 b), which corresponds to higher levels of mosquito control in the eastern region of the Municipality, where the population density is highest. Here *g* *a* *u* *s* *s*(*m*,*s* *d*,*x*) is the probability density function in *x* of the normal distribution function with mean *m* and standard deviation *sd*. Set the transmission parameters *σ*_1_(*x*,*y*)=0.00000049, *σ*_2_(*x*,*y*)=0.78 (we assume that individual mosquitoes bite multiple people, people receive multiple bites, and the probability of infection of mosquitoes is much higher than the probability of infection of people).

The diffusion terms for the infected people, uninfected mosquitoes, and infected mosquitoes in the model are understood as idealizations of the indirect spatial spread of the Zika virus infection agent. The spatial spread of the virus is dependent on the direct spread of infected people and uninfected/infected mosquitoes. The spatial movement of people in an urban setting is extremely complex, and a major challenge for epidemic modeling. We set the infected people diffusion parameter *δ*_1_=0.2, which provides a simplified way of describing the movement of infected people, in the context of the epidemic, with respect to the spatial spread of the virus. We set the mosquito diffusion parameter *δ*_2_=0.2, which is consistent with an estimated adult mosquito dispersal of 30−50 *m* per day [36].

For simplicity, we assume that the mosquito life-span is independent of spatial location, and also independent of time in the season, although for some *Aedes* species, in some environments, the life-span is correlated to temperature [35]. We take the time dependent mosquito breeding function as $\beta (t,x,y) = 300.0 \, emg(t,\bar {\mu },\bar {\sigma },\bar {\lambda)}$, where *emg* is the shifted exponentially modified gaussian

$$emg(t,\bar{\mu},\bar{\sigma},\bar{\lambda)}) = \frac{\bar{\lambda}}{2} Exp\left(\frac{\bar{\lambda}}{2}(2 \bar{\mu} + \bar{\lambda} \bar{\sigma}^{2} - 2 \, t)\right) Erfc\left(\frac{1}{\sqrt{2} \, \bar{\sigma}} (\bar{\lambda} \bar{\sigma}^{2} + \bar{\mu} -t)\right) $$

**Fig. 3:**
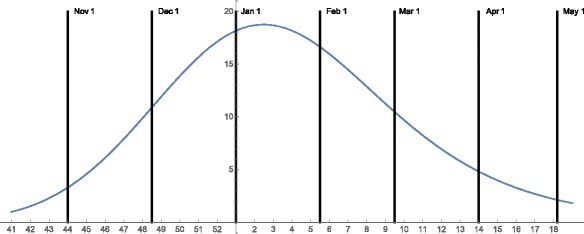
The time dependent mosquito breeding function *β*(*t*) for the 2015–2016 seasonal mosquito population in Rio de Janeiro Municipality. The graph of *β*(*t*) rises rapidly in November 2015, to its maximum in early January 2016, and then falls steadily to a low value in May 2016

Here *Erfc* is the complementary error function. The parameters are $\bar {\mu } = -2.0$, $\bar {\sigma } = 5.0$, $\bar {\lambda } = 0.2$. The graph of the seasonal mosquito breeding function *β*(*t*) is given is Fig. 3. The assumptions on the parameters of the mosquito population yield a very rapidly rising population at the beginning of the season, which quickly stabilizes to maximal capacity of approximately 14 million, and then declines gradually to very low levels from midway through the season to the end of the season. The total mosquito population, both uninfected and infected, remains mostly uniformly spatially distributed throughout the Municipality throughout the season. The infected mosquito population spatial distribution is very similar to the spatial distribution of infected people. During much of mosquito season, the ratio of total mosquitoes to total people is approximately 2 to 1, which agrees with the ratio in [15].

We set the initial outbreaks in variable locations in the Municipality. For the initial spatial distribution of infected people we set *H*_*i*_(0,*x*,*y*)=*H*_*i*0_ *g* *a* *u* *s* *s*(*x*_0_,1.0,*x*)×*g* *a* *u* *s* *s*(*y*_0_,1.0,*y*), centered at (*x*_0_,*y*_0_). The initial number of infected people at the location (determined by *H*_*i*0_) is viewed as small and above a threshold level capable of outbreak. It includes imported cases (first order) and possibly some cases generated by first order cases (higher order).

## Results

### Simulations of the model for Rio de Janeiro

We provide four simulations of the model with initial outbreaks in different locations in the Municipality.

**Fig. 4:**
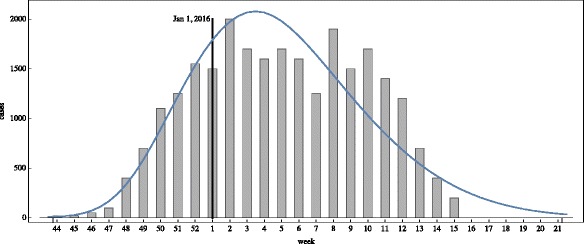
Example 1. Simulation of the reported infected cases in the Rio de Janeiro Municipality from the beginning of the epidemic season at week 44 in 2015 to week 21 in 2016 (*blue graph*). The reported case values of the simulation agree qualitatively with the number of reported cases of the Brazilian Health Ministry during this period (*grey bars*)

**Fig. 5:**
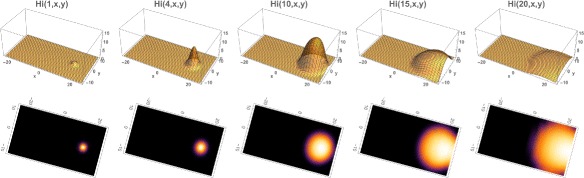
Example 1. Model simulation of the spatial distributions of infected cases in the Rio de Janeiro Municipality during the 2015–2016 epidemic season. At time 0 (November 1, 2015) a very small number of cases are located in a small region in the eastern central region of the Municipality. The spatial distributions are graphed at time=1 (week 45 in 2015), time=4 (week 49 in 2015), time=10 (week 3 in 2016), time=15 (week 8 in 2016), time=20 (week 13 in 2016). The cases concentrate in the eastern central region of the Municipality. *Top*: Density plots. *Bottom*: Heatmap plots (color magnitude scaled at each time point)

**Fig. 6:**
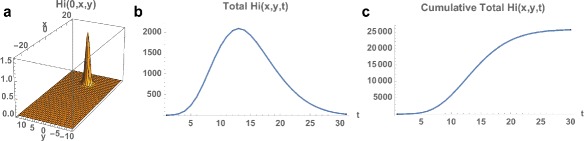
Example 1. **a** Spatial density of infected people at time *t*=0 (approximately 10). **b** The total number of infected people as a function of time. **c** The cumulative total number of infected people as a function of time, which converges to approximately 26,000 at the end of the 2015–2016 season

**Example 1**. In Example 1 the outbreak begins at time 0 on November 1, 2015 in a small eastern location of the Municipality, where *R*_0_(*x*,*y*) is very high. The total number of infected people at time 0 is 10 (*H*_*i*0_=10), with spatial distribution centered at *x*_0_=15 and *y*_0_=0, *R*_0_(15,0)≈2.27. At time 0 the total number of uninfected mosquitoes is 120,000, distributed uniformly throughout the Municipality. The total number of infected mosquitoes at time 0 is 100, with spatial distribution *V*_*i*_(0,*x*,*y*)=10.0*H*_*i*_(0,*x*,*y*). The simulation of the model (1), (4), (5) over the time period November 1, 2015 to May 21, 2016 is graphed in Figs. 4, 5 and 6. The simulation agrees qualitatively with the weekly reported case data for Rio de Janeiro Municipality in [32] (Fig. 4). The spatial distribution of infected people expands from a very small number of initial cases in a small eastern subregion of the Municipality, and disperses throughout the eastern region of the Municipality (Fig. 5). In Fig. 6 we graph the total number of infected people and the total cumulative number of infected people throughout the season. The simulation agrees qualitatively with the weekly reported case data for Rio de Janeiro Municipality given in [32], with approximately 25,500 reported cases between week 44, 2015 and week 15, 2016.

**Fig. 7:**
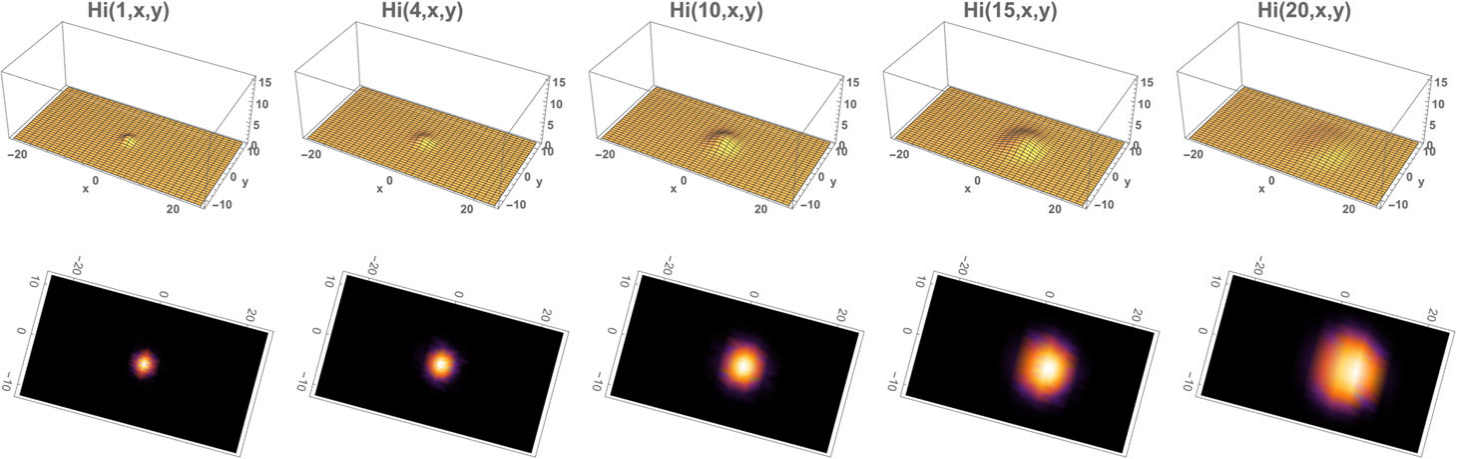
Example 2. Model simulation of the spatial distributions of infected cases in the Rio de Janeiro Municipality during the 2015–2016 epidemic season (at the same time points as in Example 1). At time 0 (November 1, 2015) a very small number of cases are located in a small region in the center of the Municipality. The cases concentrate in an eastern central region of the Municipality. *Top*: Density plots. *Bottom*: Heatmap plots (color magnitude scaled at each time point)

**Fig. 8:**
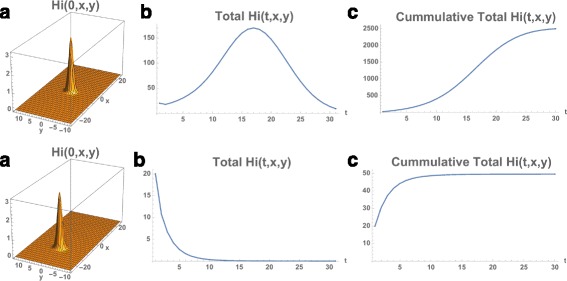
*Top*: Example 2. **a** Spatial density of infected people at time *t*=0 (approximately 20). **b** The total number of infected people as a function of time. **c** The cumulative total number of infected people as a function of time, which converges to approximately 2,500 at the end of the 2015–2016 season. *Bottom*; Example 2 modified with the initial outbreak shifted to *x*=−10 and *y*=0. The cumulative total converges to 50

**Example 2**. We repeat the simulation with the only change from Example 1 the location of the initial outbreak. We take the initial outbreak location as the center of the Municipality with *x*_0_=0 and *y*_0_=0, *R*_0_(0,0)≈1.26. The total number of infected people at time 0 is 20 (*H*_*i*0_=20). The infected population again expands from the initial location and disperses throughout the eastern region of the Municipality, but at approximately one-tenth of the number of infected cases as in Example 1 (Figs. 7 and 8). The reason is that *R*_0_(*x*,*y*) is much lower in this initial location than the initial location in Example 1, and the rise of the epidemic is much slower than in Example 1. In Fig. 8 (bottom) we repeat the example with the center of the outbreak location at *x*_0_=−10, *y*_0_=0, *R*_0_(−10,0)≈0.52. The infected cases decrease rapidly to 0, because *R*_0_(*x*,*y*) is even lower in the region of the outbreak.

**Example 3**. We again repeat the simulation with the only change from Example 1 the location of the initial outbreak. We take the initial outbreak with two locations in the center of the Municipality with

$$\begin{array}{@{}rcl@{}} 1^{st} location: & & x_{0}=0, \, y_{0}=5, \, H_{i0}=20, R_{0}(0,5) \approx 1.26,  \\ 2^{nd} location: & & x_{0}=5, \, y_{0}=-5, \, H_{i0}=10, R_{0}(5,-5) \approx 1.60.  \end{array} $$

**Fig. 9:**
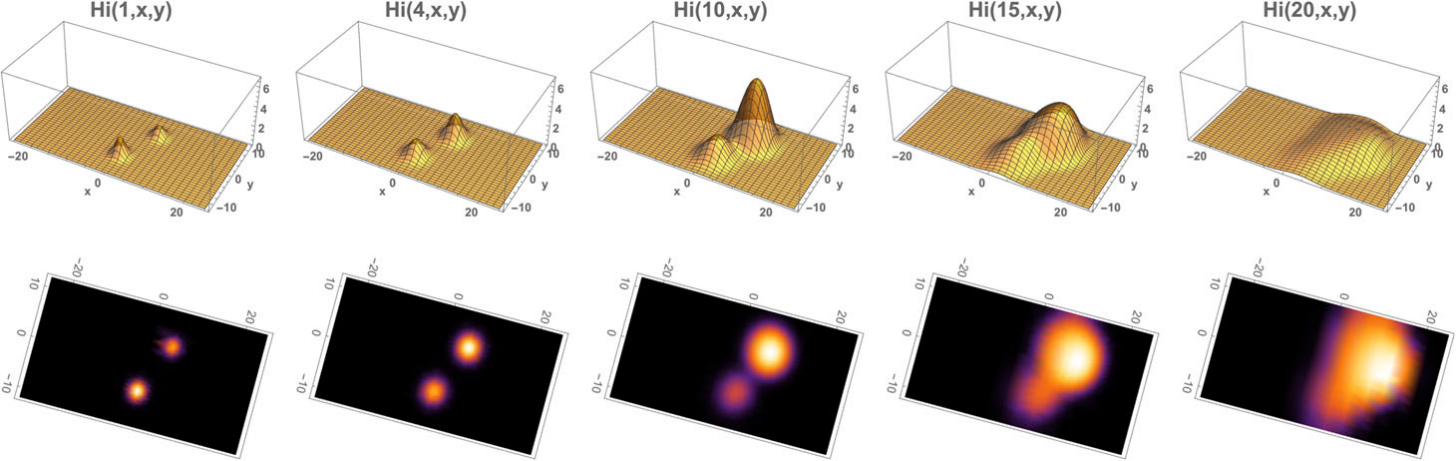
Example 3. Model simulation of the spatial distributions of infected cases in the Rio de Janeiro Municipality during the 2015–2016 epidemic season (at the same time points as in Example 1). At time 0 (November 1, 2015) a very small number of cases are located in two small regions in the center of the Municipality. The cases concentrate in the eastern region of the Municipality. *Top*: Density plots. *Bottom*: Heatmap plots (color magnitude scaled at each time point)

**Fig. 10:**
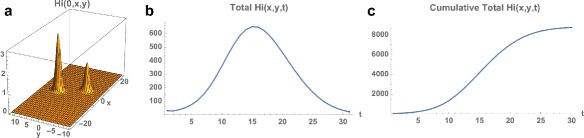
Example 3. **a** Spatial density of infected people at time *t*=0 (approximately 30). **b** The total number of infected people as a function of time. **c** The cumulative total number of infected people as a function of time, which converges to approximately 9,000 at the end of the 2015–2016 season

The total number of infected people at time 0 is 30. The infected population again expands from the initial location and disperses throughout the eastern region of the Municipality, but at approximately one-third of the number of infected cases as in Example 1 (Figs. 9 and 10). The reason is that the *R*_0_(*x*,*y*) is again lower in the initial outbreak locations than the initial outbreak location in Example 1.

**Example 4**. We again repeat the simulation with the only change from Example 1 the location of the initial outbreak. We take the initial outbreak with three locations in the eastern region of the Municipality with

$$\begin{array}{@{}rcl@{}} 1^{st} location: & & x_{0}=5, \, y_{0}=-5, \, H_{i0}=10, R_{0}(5,-5) \approx 1.60,  \\ 2^{nd} location: & & x_{0}=0, \, y_{0}=5, \, H_{i0}=30, R_{0}(0,5) \approx 1.26,  \\ 3^{rd} location: & & x_{0}=15, \, y_{0}=5, \, H_{i0}=5, R_{0}(15,5) \approx 2.16.  \end{array} $$

**Fig. 11:**
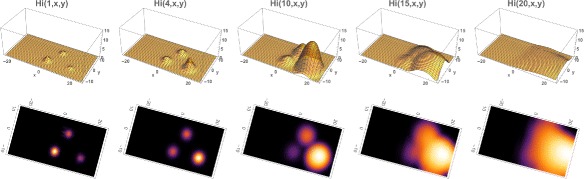
Example 4. Model simulation of the spatial distributions of infected cases in the Rio de Janeiro Municipality during the 2015–2016 epidemic season (at the same time points as in Example 1). At time 0 (November 1, 2015) a very small number of cases are located in three small eastern regions in the Municipality. The cases concentrate in the northeastern region of the Municipality. *Top*: Density plots. *Bottom*: Heatmap plots (color magnitude scaled at each time point)

**Fig. 12:**
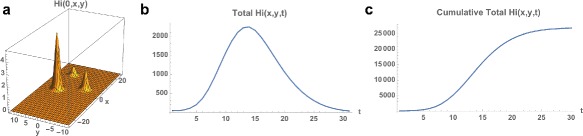
Example 4. **a** Spatial density of infected people at time *t*=0 (approximately 30). **b** The total number of infected people as a function of time. **c** The cumulative total number of infected people as a function of time, which converges to approximately 26,000 at the end of the 2015–2016 season (the same number as in Example 1.)

The total number of infected people at time 0 is 45. The infected population again expands from the initial locations and disperses throughout the eastern region of the Municipality, with the number of total cumulative infected cases the same as in Example 1 (Figs. 11 and 12). The reason is that *R*_0_(*x*,*y*) is high in the third location, as it is in the initial location in Example 1.

**Fig. 13:**
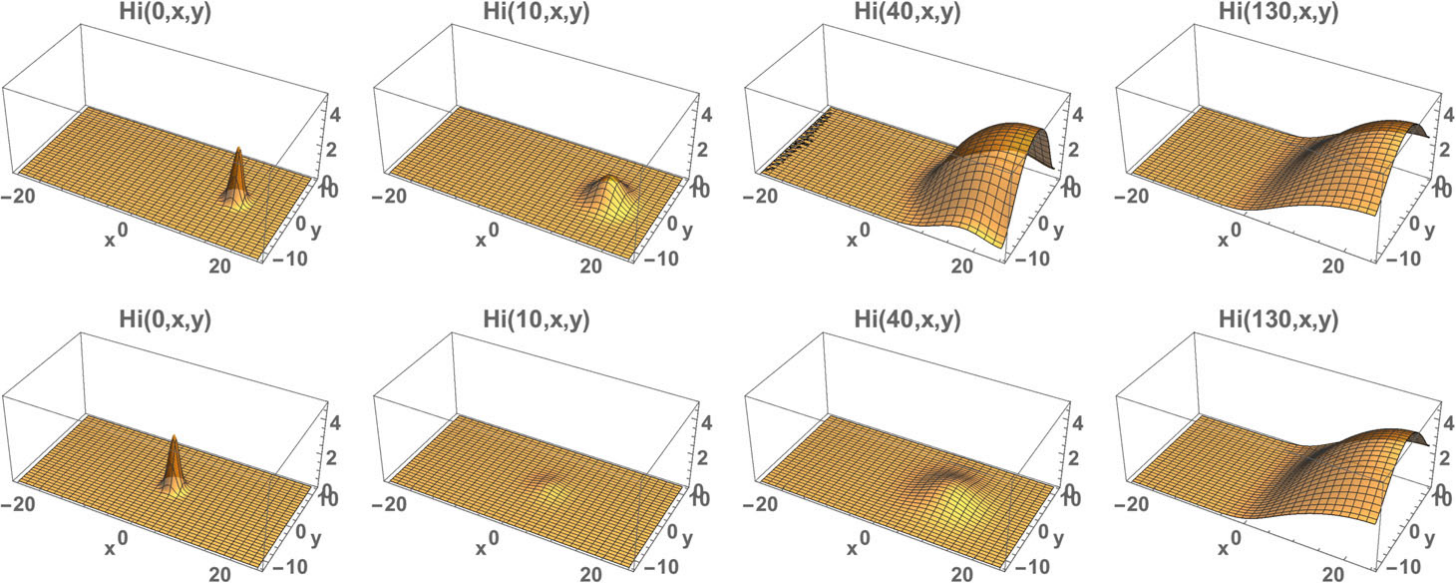
Example 5. *Top*: Model simulation of the spatial distributions of infected cases at time=0, 10, 40, 130, with the initial data located in the eastern region. *Bottom*: Model simulation of the spatial distributions of infected cases at time=0, 20, 40, 130, with the initial data located in the western region. Both simulations converge to the same limiting density, but the one with the initial data in the western region first decreases before increasing and converging

**Fig. 14:**
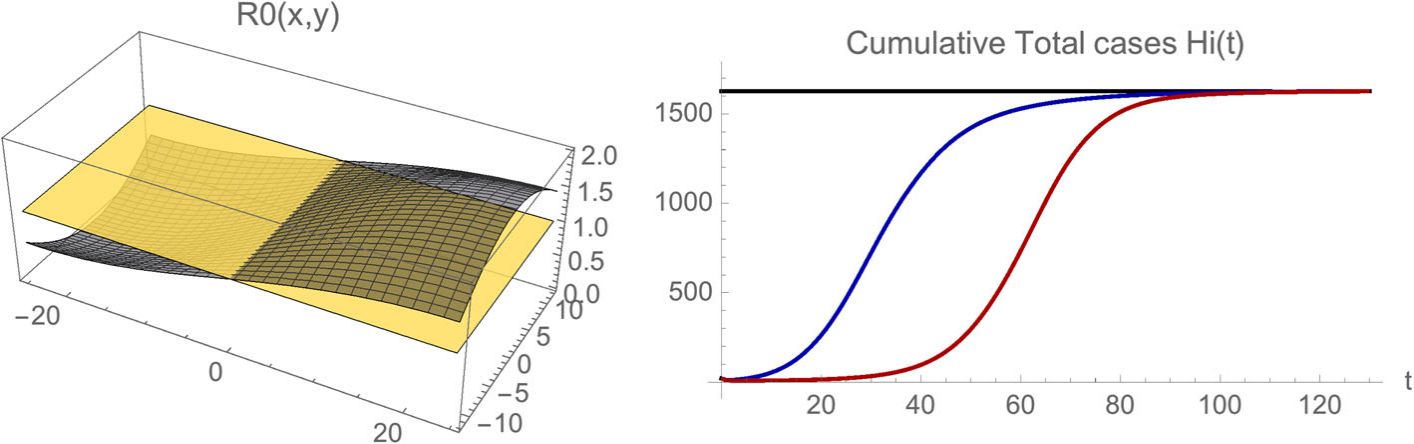
Example 5. *Left*: The spatially dependent net reproduction number *R*_0_(*x*,*y*). The average value of *R*_0_(*x*,*y*) over the whole spatial region is ≈0.984. *Right*: The cumulative total number of infected cases in the whole region as a function of time, which for both initial data in the eastern region (*blue*) and initial data in the western region (*red*) converge to ≈1,630

**Example 5**. We provide a simulation of the model Eqs. (1), (2), (3) to illustrate that the solutions may approach an endemic steady state even if the average value of *R*_0_(*x*,*y*)<1 in the spatial domain. We use the same parameters as in Rio de Janeiro Municipality, except that *σ*_1_(*x*,*y*)=0.0000001, *σ*_2_(*x*,*y*)=0.1, *μ*(*x*,*y*)=0.00005(1.0+100 *g* *a* *u* *s* *s*(−20.0,10.0,*x*)×*g* *a* *u* *s* *s*(0.0,10.0,*y*)), *δ*_1_=0.1, *δ*_2_=0.3, and *β* is set at the constant value 0.5 (the mosquito population is assumed to be present year-round rather than seasonal). The average value of *R*_0_(*x*,*y*) in the whole region is ≈0.984. The results are illustrated in Figs. 13 and 14. For initial data in the eastern region (where *R*_0_(*x*,*y*)>1), the number of infected cases increases and converges to an endemic steady state. For initial data in the western region (where *R*_0_(*x*,*y*)<1), the number of infected cases first decreases, and then increases to the same endemic state. The simulations indicate the importance of spatial heterogeneity in epidemic models, especially for outbreak scenarios. The importation of a small number of infected cases to isolated localities, may at first dissipate in sub-regions with *R*_0_(*x*,*y*)<1, but later rise and spread to sub-regions with *R*_0_(*x*,*y*)>1, and establish endemicity in the greater geographical region.

## Discussion and conclusions

The model (1), (2), (3) describes criss-cross vector-host transmission dynamics of an epidemic outbreak in a geographical region *Ω*, where the vector population is present year-round. The outbreak occurs with a small number of infected hosts in a small subregion of the much larger geographical region *Ω*. The diffusion terms describe the on-going average spatial spread of the disease microbial agent within infected vectors and infected hosts in the geographical region. The focus of the model is to describe the geographical spread from an initial localized immigration into the region, in terms of the epidemiological properties of the outbreak vector-host transmission dynamics.

We prove that the partial differential equations model (1), (2), (3) is mathematically well-posed, and compare its properties to an analogous ordinary differential equations model in the spatially independent case (Appendix). The outcomes of the model depend on the spatially distributed local reproduction number *R*_0_(*x*,*y*). In the case of year-round vector settings, simulations indicate that the connection of *R*_0_(*x*,*y*) to the outcome of an outbreak is as follows: if *R*_0_(*x*,*y*)<1 everywhere in *Ω*, then the epidemic will extinguish; if *R*_0_(*x*,*y*)>1 in some subregion of *Ω*, then the epidemic has the possibility to spread from an initial outbreak to an endemic equilibrium in *Ω*, even if the average value of *R*_0_(*x*,*y*)<1 throughout all of *Ω*.

The model Eqs. (1), (2), (3) are modified to incorporate seasonality of the vector population in Eqs. (1), (4), (5), and applied to the 2015–2016 Zika outbreak in Rio de Janeiro Municipality. Simulations of the model (Examples 1 and 4) provide qualitative agreement with the reported case data in the Municipality [32]. We argue that the assumption of an unchanging number for the susceptible population is reasonable for the Zika outbreak in Rio de Janeiro Municipality. The justification for this assumption is based on current demographic data for the Municipality [37]. Between 2010 and 2016 the population increased from approximately 6.000,000 at approximately 0.49% per year. The total number of reported cases during the 2015–2016 outbreak is less than 1% of the susceptible population, which is not significantly depleted during the outbreak.

A limitation of our model is the difficulty of estimating the number of unreported cases, and in some examples of Zika epidemics the ratio of reported cases to unreported cases has been quite high. In one study, the Federated States of Micronesia in 2007, the number of reported cases was 108 and the number of unreported cases (estimated through seroconversion testing) was estimated at 74% of the total population of 7,391 [38]. In another study, the French Polynesia outbreak in 2013–2014, the number of reported cases was estimated at 7–17% of the total number of infections, with 94% of the total population infected [33]. The setting for Rio de Janeiro Municipality is very different, however, and the demographic changes in Rio de Janeiro Municipality in one year could off-set a relatively higher ratio of unreported-to-reported cases, given that the reported cases represented approximately 0.4% of the population [31, 32]. Additionally, the probability of Zika re-infection is not yet fully known. Whether Zika could become established as an endemic disease in a larger urban population thus remains unclear [33]. Our model simulations are based on the number of reported cases, but we note that if the ratio of unreported to reported cases is significantly higher, then the parameters must be adjusted.

A limitation of our model is that it does not take into account the possibility of sexual transmission of Zika. It is noted in [2], however, that sexual transmission is a small percentage of total transmission, and may not initiate or sustain an outbreak. Another limitation of our model is that we assume the uninfected mosquito population is uniformly geographically distributed at the beginning of the season, since there is no detailed temporal geographic mosquito data available for Rio de Janeiro Municipality. We note that current investigations are developing such data for geographical regions, which could be implemented eventually for spatial models of vector borne epidemics as described by our model. One such investigation is Project Premonition [39], developed by Microsoft to autonomously locate, robotically collect, and computationally analyze mosquito populations for pathogenicity in geographical environmental regions.

The model simulation suggests that the Zika epidemic in Rio de Janeiro Municipality may rise each season from initial outbreak locations, with very small numbers of infected people, and spread through a larger region of the Municipality. Although the epidemic subsides at the end of the season, the final size of the epidemic at the end of the season depends on the initial outbreak locations of infected cases in the region, when geographic heterogeneity and time-limited seasonality are taken into account. The local reproduction number *R*_0_(*x*,*y*) indicates that the most effective interventions decrease the infection rates *σ*_1_(*x*,*y*), *σ*_2_(*x*,*y*), increase the isolation of infected people *λ*(*x*,*y*), increase the mosquito removal rate *μ*(*x*,*y*), and control the importation of infected people, all concentrated in regions of high density population *H*_*u*_(*x*,*y*) and in the beginning of the season.

For the Zika epidemic in Rio de Janeiro Municipality the model suggests that the outbreak in the 2015–2016 season will occur again in the 2016–2017 season, and in future seasons. The importation of infected cases into the Municipality at the beginning of the season is inevitable, because of the general influx of people into this major metropolitan center of Brazil. Some of these cases will not generate a further spread of cases, but some will, with consideration of spatially variable factors. The reduction of future, and more extensive, seasonal outbreaks of Zika in the Municipality requires higher level monitoring of the people arriving in the region and higher level mosquito control measures throughout the region, again with consideration of spatially variable factors.

## Appendix

### Well-posedness of the model

**Theorem.** Let *Ω* be a bounded domain in *R* ^2^ with smooth boundary *∂* *Ω* such that *Ω* lies locally on one side of *∂* *Ω*. Let *β*, *μ*, *λ*, *σ*_1_, $\sigma _{2}, \delta _{1}, \delta _{2} \, \in C_{+}^{0}(\overline {\Omega })$, and let $H_{u}, I_{0}, V_{u0},V_{i0} \in C_{+}^{1}(\overline {\Omega })$. There exists a unique global classical solution $\{H_{i}(t),V_{u}(t),V_{i}(t)\} \in C_{+}^{1}(\overline {\Omega }), \, t \geq 0$, to (1), (2), (3), satisfying boundary conditions

$$\frac{\partial}{\partial \eta} H_{i}(t,x,y) = 0, \, \frac{\partial}{\partial \eta} V_{u}(t,x,y) = 0, \, \frac{\partial}{\partial \eta} V_{i}(t,x,y) = 0, \, (x,y) \in \partial \Omega, \, t >0 $$

and initial conditions

$$H_{i}(0,x,y) = H_{i0}(x,y), \, V_{u}(0,x,y) = V_{u0}(x,y), \, V_{i}(0,x,y) = V_{i0}(x,y), \, (x,y) \in \Omega. $$

**Proof.** We first observe that a unique classical solution {*H*_*i*_(*t*),*V*_*u*_(*t*),*V*_*i*_(*t*)} exists in $C^{1}(\overline {\Omega })$ on a maximal interval of existence [0,*T*_*max*_) [40–42]. Standard arguments [42] guarantee that {*H*_*i*_(*t*),*V*_*u*_(*t*),*V*_*i*_(*t*)} remain nonnegative for *t*∈[0,*T*_*max*_). Moreover, the classical solution can be globally defined if we can establish uniform a priori bounds. Set *M*(*t*,*x*,*y*)=*V*_*u*_(*t*,*x*,*y*)+*V*_*i*_(*t*,*x*,*y*) and add Eqs. (2) and (3) to obtain

6 $$\begin{array}{@{}rcl@{}} \frac{\partial}{\partial t} M(t,x,y) &=& \nabla \cdot \delta_{2}(x,y) \nabla M(t,x,y)  \\ &&+ \, \, \beta(x,y) M(t,x,y)\, \, - \, \, \mu(x,y) M(t,x,y)^{2}.  \end{array} $$

Theorem 1 in [24] guarantees the existence of a unique global classical solution $M(t) \in C_{+}^{1}(\overline {\Omega })$ to Eq. (6) satisfying

$$\frac{\partial}{\partial \eta} M(t,x,y) = 0, \, (x,y) \in \partial \Omega, \, t \geq 0, \, M(0,x,y) = V_{u0}(x,y) + V_{i0}(x,y), \, (x,y) \in \Omega. $$

Further, in [24] it is proved that there exists $\overline {M} \in C_{+}^{0}(\overline {\Omega })$, $\overline {M} \neq 0$, such that ${\lim }_{t \rightarrow \infty }M(t) = \overline {M} \in C_{+}^{0}(\overline {\Omega })$. We note that the disease free equilibrium of (1), (2), (3) is $(0,\overline {M},0)$. From [24] there exists *N*_1_>0 such that ${max}_{t \geq 0} \| M(t) \|_{C_{+}^{0}(\overline {\Omega })} < N_{1}$, which implies $\| V_{i}(t) \|_{C_{+}^{0}(\overline {\Omega })}, \, \| V_{u}(t) \|_{C_{+}^{0}(\overline {\Omega })} < N_{1}$. Then, since *λ*>0 in (1), there exists *N*_2_>0 such that $\| H_{i}(t) \|_{C_{+}^{0}(\overline {\Omega })} < N_{2}$. Consequently, the solution exists globally on [0,*∞*).

### The model equations without spatial dependence

The Eqs. (1), (2), (3) without spatial dependence are

7 $$\begin{array}{@{}rcl@{}}  \quad \frac{d}{d t} H_{i}(t) &=& - \lambda H_{i}(t) + \sigma_{1} \, V_{i}(t) \, H_{u}  \end{array} $$

8 $$\begin{array}{@{}rcl@{}} \frac{d}{d t} V_{u} (t) &=&\beta (V_{u}(t) + V_{i}(t)) -\sigma_{2} V_{u}(t) H_{i}(t) - \mu (V_{u}(t) + V_{i}(t)) V_{u}(t)  \end{array} $$

9 $$\begin{array}{@{}rcl@{}} \frac{d}{d t} V_{i} (t) &=& \sigma_{2} V_{u}(t) H_{i}(t) - \mu (V_{u}(t) + V_{i}(t)) V_{i}(t)  \end{array} $$

with initial conditions *H*_*i*_(0)=*H*_*i*0_, *V*_*u*_(0)=*V*_*u*0_, *V*_*i*_(0)=*V*_*i*0_. Set the basic reproduction number *R*_0_=*H*_*u*_ *σ*_1_ *σ*_2_/*λ* *μ*. We note that *R*_0_ is independent of the vector reproduction rate *β*. The epidemic size of the epidemic, however, is proportional to *β*, as seen in their formulas below. The behavior of solutions of Eqs. (7), (8), (9) can be classified as follows:

**Proposition** If *R*_0_<1, then the only steady states of (7), (8), (9) in $R_{+}^{3}$ are *s* *s*_0_=(0,0,0), which is unstable in $R_{+}^{3}$, and *s* *s*_1_=(0,*β*/*μ*,0), which is proportional to *β* and locally exponentially asymptotically stable in $R_{+}^{3}$. If *R*_0_<1, *H*_*i*_(0)>0, and *V*_*i*_(0)=0, then (*H*_*i*_(*t*),*V*_*u*_(*t*),*V*_*i*_(*t*)) converges to $(0,\overline {M},0)$. If *R*_0_>1, then *s* *s*_0_ and *s* *s*_1_ are unstable in $R_{+}^{3}$ and there is another steady state in $R_{+}^{3}$,

$${ss}_{2} = \left(\frac{\beta (H_{u} \sigma_{1} \sigma_{2} -\lambda \mu)}{\lambda \mu \sigma_{2}}, \frac{\beta \lambda}{H_{u} \sigma_{1} \sigma_{2}}, \frac{\beta (H_{u} \sigma_{1} \sigma_{2} -\lambda \mu)}{H_{u} \mu \sigma_{1} \sigma_{2}} \right)$$

$$= \left(\frac{\beta(R_{0} -1)}{\sigma_{2}}, \frac{\beta}{R_{0} \mu}, \frac{\lambda \beta (R_{0} - 1)}{ H_{u} \sigma_{1} \sigma_{2}} \right).$$

which is proportional to *β* and locally exponentially asymptotically stable in $R_{+}^{3}$.

**Proof.** Set *M*(*t*)=*V*_*u*_(*t*)+*V*_*i*_(*t*) and $\overline {M} = \beta / \mu $. Then *M* ^′^(*t*)=*β* *M*(*t*)−*μ* *M*(*t*)^2^ and ${\lim }_{t \rightarrow \infty } M(t) = \overline {M}$. It can be verified that the steady states of (7), (8), (9) in $R_{+}^{3}$ are *s* *s*_0_,*s* *s*_1_, and *s* *s*_2_. The Jacobian of (7), (8), (9) at *s* *s*_0_ is

$$J(0,0,0) = \left[ \begin{array}{ccc} -\lambda & 0 & H_{u} \sigma_{1} \\ 0 & \beta & \beta \\ 0 & 0 & 0 \end{array} \right]$$

with eigenvalues {−*λ*,*β*,0}, which means that (0,0,0) is unstable. If *H*_*i*_(0)>0 and *V*_*i*_(0)=0, then (7) implies $H_{i}^{\prime }(0) < 0$. Assume there is a smallest positive time *t* ^∗^ such that $H_{i}^{\prime }(t^{\ast }) = 0$. Then (7) implies *H*_*i*_(*t* ^∗^)=(*σ*_1_ *H*_*u*_/*λ*)*V*_*i*_(*t* ^∗^). If *R*_0_<1, then (9) implies

$$V_{i}^{\prime}(t^{\ast}) = \frac{\sigma_{1} \sigma_{2} H_{u}}{ \lambda} \, V_{i}(t^{\ast}) (M(t^{\ast}) - V_{i}(t^{\ast})) - \mu V_{i}(t^{\ast}) M(t^{\ast}) < - \frac{\sigma_{1} \, \sigma_{2} \, H_{u}}{ \lambda} \, V_{i}(t^{\ast})^{2} < 0. $$

Then (7) implies

$$H_{i}^{\prime \prime}(t^{\ast}) = - \lambda H_{i}^{\prime}(t^{\ast}) + \sigma_{1} H_{u} V_{i}^{\prime}(t^{\ast}) < 0, $$

which implies *H*_*i*_(*t*) is strictly decreasing at *t* ^∗^, yielding a contradiction. Thus, *H*_*i*_(*t*) is strictly decreasing for all *t*≥0. Let $H_{i,\infty } = {\lim }_{t \rightarrow \infty } H_{i}(t) \geq 0$. Assume *H*_*i*,*∞*_>0. Then (9) implies *l* *i* *m*_*t*→*∞*_ *V*_*i*_(*t*)=*λ* *H*_*i*.*∞*_/*σ*_1_ *H*_*u*_>0. Equation (8) then implies ${\lim }_{t \rightarrow \infty }V_{u}(t) = \beta \overline {M} / (\sigma _{2} H_{i,\infty } + \mu \overline {M})$. Then $(H_{i,\infty },\beta \overline {M} / (\sigma _{2} H_{i,\infty } + \mu \overline {M}),\lambda H_{i,\infty } / (\sigma _{1} H_{u}))$ is a steady state of (7), (8), (9). If *R*_0_<1, then *H*_*i*,*∞*_=0, yielding a contradiction. Thus, *H*_*i*,*∞*_=0.

The eigenvalues of the Jacobian of (7), (8), (9) at *s* *s*_1_

$$J(0,\beta / \mu,0) = \left[ \begin{array}{ccc} -\lambda & 0 & H_{u} \sigma_{2} \\ - \beta \sigma_{1} / \mu & - \beta &0 \\ \beta \sigma_{1} / \mu & 0 & - \beta \end{array} \right]$$

are

$$\{- \beta, \frac{- \beta - \lambda - \sqrt{(\beta - \lambda)^{2} + 4 R_{0} \beta \lambda}}{2}, \frac{- \beta - \lambda + \sqrt{(\beta - \lambda)^{2} + 4 R_{0} \beta \lambda}}{2}\}.$$

Thus, *J*(0,*β*/*μ*,0) is unstable if *R*_0_>1 and locally exponentially asymptotically stable if *R*_0_<1.

The Jacobian of (7),(8),(9) at *s* *s*_2_ is

$$\left[ \begin{array}{ccc} - \lambda & 0 & H_{u} \sigma_{1} \\ - \frac{\beta \lambda}{H_{u} \sigma_{1}} & \beta(1- \frac{\lambda \mu}{H_{u} \sigma_{1} \sigma_{1}} - \frac{H_{u} \sigma_{1} \sigma_{2}}{\lambda \mu}) & \beta (1 - \frac{\lambda \mu}{H_{u} \sigma_{1} \sigma_{2}}) \\ \frac{\beta \lambda}{H_{u} \sigma_{1}} & \beta(- 2 + \frac{\lambda \mu}{H_{u} \sigma_{1} \sigma_{2}} +\frac{H_{u} \sigma_{1} \sigma_{2}}{\lambda \mu}) & \beta (- 2 + \frac{\lambda \mu}{H_{u} \sigma_{1} \sigma_{2}}) \end{array} \right] $$

$$= \left[ \begin{array}{ccc} - \lambda & 0 & \frac{R_{0} \lambda \mu}{\sigma_{2}} \\ - \frac{\beta \sigma_{2}}{R_{0} \mu} & \beta(1- \frac{1}{R_{0}} - R_{0}) & \beta (1 - \frac{1}{R_{0}}) \\ \frac{\beta \sigma_{2}}{R_{0} \mu} & \beta(- 2 + \frac{1}{R_{0}} + R_{0}) & \beta (- 2 + \frac{1}{R_{0}}) \end{array} \right]. $$

with eigenvalues

$$\{- \beta, \frac{- R_{0} \beta - \lambda - \sqrt{(R_{0} \beta - \lambda)^{2} + 4 \beta \lambda}}{2}, \frac{- R_{0} \beta - \lambda + \sqrt{(R_{0} \beta - \lambda)^{2} + 4 R_{0} \beta \lambda}}{2}\}. $$

Since −(*R*_0_ *β*+*λ*)^2^+(*R*_0_ *β*−*λ*)^2^+4*β* *λ*=−4(*R*_0_−1)*β* *λ*<0 if *R*_0_>1, the eigenvalues of the Jacobian at *s* *s*_2_ are strictly negative if *R*_0_>1, which means that *s* *s*_2_ is locally exponentially asymptotically stable if *R*_0_>1.
